# Comparison of 2D, 3D *In Vitro*, and *Ex Vivo* Platforms for Modeling the Rat Small Intestine

**DOI:** 10.3390/bioengineering13030349

**Published:** 2026-03-17

**Authors:** Shani Elias-Kirma, Reece McCoy, Douglas van Niekerk, Verena Stoeger, Sophie Oldroyd, Emma Sumner, Achilleas Savva, Róisín M. Owens

**Affiliations:** 1 Department of Chemical Engineering and Biotechnology, University of Cambridge, Philippa Fawcett Drive, Cambridge CB3 0AS, UK; rm991@cam.ac.uk (R.M.); dcv24@cam.ac.uk (D.v.N.); vs506@cam.ac.uk (V.S.); sophieoldroyd@gmail.com (S.O.); a.savva@tudelft.nl (A.S.); 2 Department of Microelectronics, Faculty of Electrical Engineering, Mathematics and Computer Science, Delft University of Technology, Mekelweg 4, 2628 CD Delft, The Netherlands

**Keywords:** 3D in vitro, bioelectronics, PEDOT:PSS scaffold, rat, small intestine barrier

## Abstract

Physiologically relevant in vitro intestinal models are essential for studying key physiological processes, including barrier function, drug screening and gut-microbiota interactions. However, conventional 2D culture systems often fail to recapitulate structural and functional complexity. Here, we aimed to validate a 3D bioelectronic transmembrane platform, previously used for monitoring human intestinal epithelium and vascular endothelium, for modeling the rat small intestinal barrier in vitro. The device integrates a poly(3,4-ethylenedioxythiophene):poly(styrenesulfonate) (PEDOT:PSS) scaffold supporting co-cultures of rat intestinal epithelial cells (IEC-6) and rat fibroblasts (208F), enabling real-time monitoring of barrier formation through electrical measurements using electrochemical impedance spectroscopy (EIS). Barrier formation was monitored over 21 days and exhibited a time-dependent increase in barrier resistance. The 3D platform was compared with traditional 2D insert-based cultures and ex vivo rat tissue using an Ethylene Glycol Tetraacetic Acid (EGTA)-induced calcium switch assay to evaluate barrier disruption and recovery. EGTA treatment and removal induced reversible barrier disruption in the 3D in vitro and ex vivo models, whereas 2D in vitro cultures showed limited recovery. These findings demonstrate that the 3D platform more faithfully recapitulates native tissue architecture and function, closely paralleling ex vivo responses. Our study highlights the importance of validating advanced 3D in vitro models and establishes this bioelectronic platform as a robust tool for drug screening, barrier studies, and preclinical gastrointestinal research.

## 1. Introduction

The small intestine plays a central role in nutrient absorption, immune regulation, and barrier function, making it a key target for studies in physiology, drug screening, gut–microbiota interactions and disease modeling [1,2,3,4]. To investigate intestinal mechanisms, researchers have traditionally relied on in vitro cell culture platforms [5,6,7] and in vivo animal models [3,8,9]. However, despite many promising outcomes in preclinical animal studies, a large proportion of drug candidates ultimately fail in clinical trials [10,11]. This discrepancy highlights the critical anatomical, immunological, and physiological differences between animal models and humans [12,13]. Consequently, there is an urgent need for more physiologically relevant human-based in vitro platforms that can overcome these limitations and enable the study of intestinal cellular mechanisms in a controlled environment [14].

Traditional in vitro assays mainly rely on 2D cell cultures, which are relatively easy to maintain but fail to replicate the complex 3D microenvironment and architecture of native intestinal tissue [7]. In contrast, 3D culture platforms provide a more physiologically relevant context and more closely mimic the in vivo tissue organization and function. Over the past decade, diverse in vitro 3D intestinal models have been developed, including organoids [15], *organ-on-chip* platforms [16,17] and scaffold-based systems [18,19,20,21]. For instance, Sato et al. [15] demonstrated that organoids derived from stem cell cultures can recapitulate intestinal architecture and function, while Inui et al. [22] developed functional intestinal monolayers from human induced pluripotent stem cells (iPSCs)-derived organoids, enhancing drug-metabolizing enzyme and transporter expression, thus improving the model’s utility for drug discovery and pharmacological research.

*Organ-on-chip* platforms [17] have further enabled dynamic in vitro modeling of the intestinal microenvironment, incorporating mechanical cues and host-microbiota interactions. For example, Kim and Ingber [16] showed that physical forces, such as peristalsis-like motions and fluid flow, play a key role in guiding tissue development in vitro. Ewart et al. [11] emphasized the importance of validating in vitro platforms by employing the Emulate Human Liver-Chip to assess drug-induced liver injury. The study evaluated the platform’s performance against spheroid and animal model data and assessed its economic impact on drug development, confirming the platform’s reliability as a qualified preclinical toxicology model.

Recent advances in biomaterial-based scaffold systems, such as custom-engineered hydrogel bioinks, like the ECM-DNA-CPO bionic matrix [23], have enabled the creation of organoid-like constructs and bioactive scaffolds that support tissue-specific differentiation and bone regeneration. These advances highlight the importance of engineered scaffolds in next-generation tissue engineering and disease modeling [23,24].

Notably, scaffold-based systems, including those based on the conducting polymer PEDOT:PSS, have been developed to provide structural support and offer a unique advantage, enabling real-time and non-invasive monitoring of electrical activity in 3D tissue models. These scaffolds have been applied for 3D in vitro tissue engineering in various human tissues, including the intestine [18,19], bone [25], brain tissue [26] and cardiac tissue [27].

More specifically, electroactive PEDOT:PSS scaffolds can be integrated into transistors [20,28] or electrodes [18], allowing for quantitative electrical readouts of 3D cell behavior through standard measurements. Such platforms have been used to monitor cell adhesion, growth, and barrier formation in real time [19]. Moreover, bioelectronic systems with tissue-like complexity, including 3D models, highlight the potential of PEDOT:PSS devices as reliable complementary tools alongside animal models for disease modeling, drug screening, and tissue engineering [29]. Expanding this concept further, Pitsalidis et al. [18] developed an organic electronic transmembrane (e-transmembrane) device based on PEDOT:PSS scaffolds, designed to host and monitor 3D cell cultures in real time. Initially, this platform enabled quantitative monitoring of barrier formation in models of human intestinal epithelium and vascular endothelium, using Caco-2 co-cultured with HT29-MTX cells and HUVEC cells, respectively. To reconstruct epithelial and endothelial monolayers, human fibroblasts were incorporated within the porous scaffold to provide structural support and create a favorable microenvironment for the subsequent seeding of other tissue types. In a follow-up study, Moysidou et al. [19] expanded the use of the 3D e-transmembrane device and examined host–microbe interactions, using intestinal co-cultures of Caco-2 and HT29-MTX cells with human fibroblasts. Additionally, Stoeger et al. [21] applied the e-transmembrane device co-cultured with Caco-2 and HT29-MTX cells to explore the effects of butyrate on intestinal absorption and nutrient transport. Furthermore, Savva et al. [20] employed a PEDOT:PSS 3D electrode and showed the ability to control the scaffold’s electrical signature via processing additives (i.e., DBSA (sodium dodecylbenzenesulfonate)) and crosslinkers (i.e., GOPS and PEGDE) ((3-glycidyloxypropyl)trimethoxysilane and poly(ethylene glycol) diglycidyl ether, respectively) to maximize the sensitivity in monitoring cell proliferation and differentiation. Inspired by this approach, in the present study, the DBSA percentage in the mixture was adjusted, and PEGDE was used as a crosslinker to create a scaffold suitable for cell seeding and capable of detecting barrier resistance, as discussed in the Supplementary Information in Figure S1. Similarly, Barberio et al. [26] explored PEDOT:PSS-ECM scaffolds (a mixture of collagen, hyaluronic acid, and laminin) for human neuronal cell culture. Their findings suggest these scaffolds can support the growth of functional neurons, making them suitable for neuronal tissue engineering applications. In a later work, Furlani et al. [27] used PEDOT:PSS–gelatin scaffolds that mimic the mechanical properties of native cardiac tissue and support the growth of cardiac cells, indicating their potential for heart tissue engineering. Collectively, these studies demonstrate the broad applicability of conductive polymer-based scaffolds for engineering functional 3D tissues while enabling electrical interrogation of cellular behavior. To fully realize the potential of such 3D bioelectronic systems, rigorous validation is required against both conventional in vitro models and relevant ex vivo or in vivo tissues, ensuring that their functional readouts accurately reflect physiological conditions [11,30].

While the 3D models described above primarily use human-derived cells to replicate key aspects of human tissue, their validation in the laboratory often benefits from the complementary use of animal-based systems [6]. Rodent models remain highly valuable due to their accessibility, reproducibility, and compatibility with existing in vivo datasets [31], and are frequently used to investigate gut–microbiota interactions [32] and paracellular permeability [33]. In this context, rat-derived models serve as a practical intermediate step, allowing researchers to evaluate and validate the performance of newly developed human-based devices and co-culture platforms before translating findings into more complex human-derived models [32].

Motivated by the growing need for physiologically relevant 3D intestinal models that can complement animal experiments in fields such as drug screening, we aimed to validate a 3D electronic transmembrane device and compare its performance with ex vivo conditions. Here, we present the validation of a previously established 3D e-transmembrane device designed for real-time monitoring of electrical signaling in in vitro models of human intestinal epithelium and vascular endothelium [18,19]. This study extends its application to a rat small intestine model, enabling direct comparison with a traditional insert-based 2D in vitro system using a well-established barrier disruption assay with EGTA and three complementary electrical measurement approaches tailored to each specific platform: a voltohmmeter for the 2D emphasized the critical importance of validating in vitro platforms by employing the Emulate Human Liver-Chip to assess drug-induced liver injury system, a potentiostat for the 3D system, and a Ussing chamber for the ex vivo setup. Our findings demonstrate the improved capability of 3D platforms to more faithfully reproduce native tissue architecture and function.

In contrast to many existing 3D intestinal models, such as organoids or conventional scaffold-based cultures that rely mainly on endpoint biochemical or imaging-based analyses [7], the presented bioelectronic platform enables continuous and non-invasive electrical monitoring of epithelial barrier formation and integrity in real time. This platform enables the reproduction of physiological electrical signaling and barrier responses in a rat small intestine model, providing a functional readout of tissue behavior and supporting dynamic assessment of barrier integrity. These features offer a significant advantage for applications in drug screening, toxicity testing, and mechanistic studies.

In summary, the validated 3D bioelectronic platform presented here offers a promising bridge between conventional animal models and next-generation in vitro systems. Importantly, rigorous validation of 3D in vitro devices against biological benchmarks—such as ex vivo and in vivo models—is essential for their reliable integration into translational research pipelines.

## 2. Materials and Methods

### 2.1. Cell Culture

#### 2.1.1. Cell Culture Maintenance

In this study, two types of cells were used: IEC-6 cells, rat small intestine epithelial cells (ECACC, 88071401), and 208F cells, a rat fibroblast cell line (ECACC, 85103116). Both cells were cultured under immersed conditions in a tissue culture T75-flask (TPP, 90025), with DMEM medium (Sigma-Aldrich, St. Louis, MO, USA, D5671) containing 10% fetal bovine serum (FBS) (Gibco, A5256801), 2 mM Glutamax (Thermo Scientific, Waltham, MA, USA, 35050038), 0.5% Penicillin Streptomycin—Penstrep (Thermo Scientific, Waltham, MA, USA, 15140122), and 0.1% Insulin, Human recombinant zinc (Gibco, 12585-014). The medium was changed every second or third day until cells reached ~90% confluence. A 0.05% Trypsin-EDTA solution (Gibco, 11580626) was then used to detach cells from culture dishes. The cells were separated from the medium by centrifugation (5 min, 300× *g*). The supernatant was carefully removed, and the cells were subsequently resuspended in fresh medium. Thereafter, cells were used for experiments as described in detail below. IEC-6 cells were used between passages 24 and 28, and 208F cells between passages 3 and 10. Cells were incubated at 37 °C in a humidified atmosphere containing 5% CO_2_. Mycoplasma controls were performed routinely using the MycoAlert mycoplasma detection kit (Lonza, Basel, Switzerland, BELT07-218) without exhibiting infection.

#### 2.1.2. Cell Seeding Within Insert

For 2D monoculture experiments, 1 × 10^4^ IEC-6 or 208F cells were seeded on top of a tissue culture 24-well plate insert (Greiner Bio-One, Kremsmünster, Austria, 662641). For co-culture, 1 × 10^4^ 208F cells, suspended in 200 µL of complete growth medium, were seeded on the basal side of the insert (while flipping the insert), and were incubated for 2 h at 37 °C in a humidified atmosphere containing 5% CO_2_. Next, after placing the insert inside the well and adding 300 µL media to the basal compartment, 1 × 10^4^ IEC-6 were seeded on the apical side of the insert. The culture was maintained under immersed conditions with media change every other day for 14 days before further experiments.

### 2.2. Transepithelial Electrical Resistance (TEER) Measurement

For 2D mono- and co-culture within an insert, barrier integrity was monitored by measuring TEER values using the World Precision Instrument Voltohmmeter (EVOM 3). At each time point, the TEER value was calculated as averages of n = 4–9 technical repeats per biological repeat (N), with N = 3–4 repeats, and represented as the mean ± SD for each cell group. TEER represented in [Ω·cm^2^] = (Raw value[Ω] − blank value[Ω]) ∙ 0.33 cm^2^ (which is the growth area for 24 well inserts) with 3 readings per well. After each TEER measurement, the medium was replaced.

### 2.3. 3D e-Transmembrane Device

#### 2.3.1. Scaffold Fabrication

Conducting polymer scaffolds were prepared according to our previously published study [20]. An aqueous dispersion of PEDOT:PSS (Clevios PH-1000, Heraeus, Hanau, Germany) was filtered twice with 0.8 mm filters (Sartorius, Göttingen, Germany, 16592), then was mixed with 3% (*w*/*v*)-PEGDE (Sigma-Aldrich, St. Louis, MO, USA, 475696) and was sonicated for 10 min at room temperature (RT). To optimize the scaffolds’ pore size and cell culture within the 3D device, we tested different amounts of the processing additive DBSA (Sigma-Aldrich, St. Louis, MO, USA, 44198) (i.e., 0.2% and 0.5% (*w*/*v*)). The DBSA was added to the PEDOT:PSS mixture, followed by sonication for 15 min at RT. The influence of the different amounts of DBSA on the pore architecture of the scaffold, as well as on the impedance spectra of the device, is shown in the Supplementary Materials, Figure S1. The mixture was pipetted into a 48-well plate at 500 µL per well, and was freeze-dried (VirTis Advantage Plus) as follows. First, the samples were held at 20 °C for 30 min, then were cooled to 2 °C for 35 min (0.51 °C/min) and were held at 2 °C for 15 min. Next, the freezing stage decreases the temperature of the samples from 2° to −34 °C for 40 min (0.9 °C/min) at 500 mTorr, followed by another temperature drop from −34 °C to −40 °C over 60 min and held at that temperature for 30 min. Next, in the drying phase, the temperature was kept for 2 h at 80 mTorr and increased to 20 °C over 7 h (0.15 °C/min). Finally, the scaffolds were annealed at 65 °C for 4 h to promote cross-linking. The PEDOT:PSS scaffolds were removed from the well plate and were sliced into 400 µm thickness using a LEICA VT1200S micro-vibratome with a blade speed of 0.8 mm/s and a vibration amplitude of 1 Hz.

#### 2.3.2. Device Design and Fabrication

**Figure 1:**
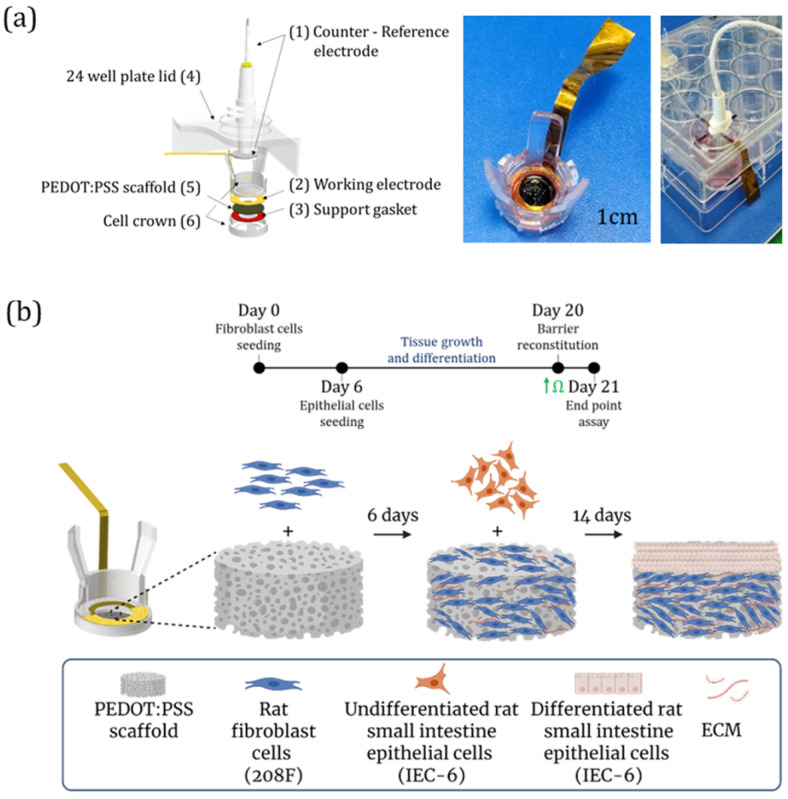
Design of the 3D e-transmembrane device and the experimental pipeline. (**a**) Computer-aided design (CAD) schematic of the 3D e-transmembrane device, showing the (1) counter–reference electrode, (2) working electrode, (3) support gasket, (4) 24-well plate lid, (5) PEDOT:PSS scaffold, and (6) cell crown. Photographs of the basal side (middle) and the fully assembled device (right). (**b**) Schematic illustration of the tissue engineering pipeline for establishing the 3D intestinal barrier model within the PEDOT:PSS scaffold. Created in BioRender. Kirma, S. (2026). BioRender.com/dlosj1w.

Following previously established protocols [18,19], the e-transmembrane devices were prepared after a modification of the counter electrode material, which was replaced with a stainless steel mesh (Inoxia, Woven Wire Mesh 325) [21]. Briefly, the device consists of two main compartments: (1) an apical compartment, which features a stainless-steel mesh counter/reference electrode integrated into a modified 24-well plate lid, and (2) a basal compartment, which houses a cell crown (Scaffdex C00006N) containing a 400 μm PEDOT:PSS scaffold. The scaffold is in contact with a ring-shaped gold sheet that extends outside the well to enable external electrical connection [18]; together, they serve as the working electrode, supported by a Kapton film gasket. A scheme and photographs of the fully assembled 3D device are shown in Figure 1a.

#### 2.3.3. Cell Seeding Within the e-Transmembrane Device

Following device fabrication and prior to cell seeding, a sterilization procedure was performed. The device (including the scaffolds within it) was soaked in 70% ethanol for a minimum of 2 h at RT. Next, the device was washed three times with sterile water, followed by three washes with PBS. Finally, the device was incubated for 2 h at 37 °C in a humidified atmosphere containing 5% CO_2_ with complete culture media. After that, cells could be seeded [18,19,21]. Briefly, 6 × 10^5^ 208F cells, suspended in 35 µL media, were seeded on the apical side of the PEDOT:PSS, as was well-established in previous work of our group [18,19]. 208F cells were maintained under immersed conditions for 5 days, allowing for a 3D network of fibroblasts and ECM development. Then on day 6, 7 × 10^5^ undifferentiated IEC-6 cells were seeded on top of the fibroblast layer (Figure 1b) using the same procedure. The co-culture was maintained under immersed conditions with media changes every other day for 14 days before further experiments (on day 21 in total).

### 2.4. Imaging

#### 2.4.1. Scanning Electron Microscopy (SEM)

Samples were washed three times with PBS and fixed in sodium cacodylate, 0.1 M buffer solution (AlfaAesar, J622021) for 60 min at RT, followed by washing three times with PBS. Next, samples were dehydrated through a graded ethanol series and further processed by critical point drying and sputter coating with carbon (17 nm). Images were acquired with a FEI Nova NanoSEM 450.

#### 2.4.2. Immunofluorescence Microscopy

Directly after cell fixation using 4% PFA, cells were treated with 0.05% Triton X-100 (Sigma-Aldrich, St. Louis, MO, USA, T8787) for 5 min at RT to increase membrane permeability and were blocked for non-specific binding using 2% BSA for 1 h at RT. For tight junction proteins, Zonula occludens-1 (ZO-1), IEC-6 cells were incubated with the primary antibodies rat anti-ZO-1 (Invitrogen, Carlsbad, CA, USA, 14977680) diluted with 2% BSA (ratio of 1:200) overnight at 4 °C, followed by incubation for 1 h at RT with secondary antibody Goat anti-rat, Alexa Fluor 647 (Thermo Fisher scientific, Waltham, MA, USA, A-21247) diluted with 2% BSA (ratio of 1:400). For Extra Cellular Matrix (ECM) proteins, Fibronectin, and for smooth muscle actin proteins (SMA), respectively, 208F cells were incubated with the primary antibodies mouse anti-Fibronectin (Abcam, Cambridge, UK, ab6328) diluted with 2% BSA (ratio of 1:200), and rabbit anti-SMA (Abcam, Cambridge, UK, ab124964) diluted with 2% BSA (ratio of 1:100), for 1 h at RT followed by incubation for 1 h at RT with secondary antibody Goat anti-mouse, Alexa Fluor 647 (Thermo Fisher scientific, Waltham, MA, USA, A-21235) and Goat anti-rabbit, Alexa Fluor 488 (Thermo Fisher scientific, Waltham, MA, USA, A-11008), diluted with 2% BSA (ratio of 1:400). For F-actin staining, cells were incubated with Phalloidin Alexa Fluor 488 (Thermo Fisher scientific, Waltham, MA, USA, A12379) diluted with PBS (ratio of 1:500) for 1 h at RT. For Hoechst nucleic acid staining, cells were incubated with Bisbenzimide Hoechst 33342 (Abcam, Cambridge, UK, ab145597), diluted with PBS (ratio of 1:1000) for 10 min at RT. After each step, cells were washed three times with PBS. Finally, confocal microscopy imaging of fluorescent immunostaining was performed using an Axio Observer Z1 LSM 800 (ZEISS, Oberkochen, Germany) confocal microscope.

### 2.5. Electrochemical Impedance Spectroscopy (EIS) and Modeling

EIS measurements were performed using a potentiostat (Metrohm Autolab), as was published in detail [18,19]. Briefly, before the medium was changed, the e-transmembrane devices were connected to the potentiostat in a two-electrode configuration. A stainless-steel mesh counter electrode (CE) was immersed in the medium and kept at a constant distance from the scaffold, which was mechanically clamped in contact with a gold electrode, acting together as a working electrode (WE), as shown in Figure 1a. It is noted here that using the potentiostat in a two-electrode configuration requires using the CE as the reference electrode (RE). EIS was performed over a frequency range from 10^5^ Hz to 0.1 Hz, recording 10 points per decade. Measurements were performed at a bias voltage of 0.1 V_{we vs. CRE}_ with an applied AC voltage amplitude of 10 mV. For further details, see [19]. Simulation and fitting of the measured electrical resistance (e.g., the barrier resistance parameter, Rb, generated by the epithelial cells) was carried out by using a bespoke MATLAB application (version 2021b), which was designed and published previously [19]. The most commonly used equivalent circuit, which will be discussed later in the manuscript, was used to fit the data.

### 2.6. Calcium Switch Assay

To examine the difference in recovery of epithelial barrier integrity disruption between insert (2D), e-transmembrane (3D) tissue culture, and ex vivo tissue in real time, a calcium switch assay was used. We incubated the cells for 30 min with 5 mM Ethylene Glycol Tetraacetic Acid (EGTA) (AlfaAesar, Ward Hill, MA, USA, J60767-AD) (diluted from stock in growth medium), then monitored the effect on barrier integrity (using EVOM, potentiostat and Ussing chamber for 2D, 3D and ex vivo tissue, respectively). The cells were then washed with medium, incubated for another 30 min, and we monitored the recovery of the epithelial barrier. All incubations were performed at 37 °C in a humidified atmosphere containing 5% CO_2_.

### 2.7. Ex Vivo Ussing Chamber

To test the effect of 5 mM EGTA on a rat small intestine tissue barrier integrity, an ex vivo ion transport experiment was conducted. A 2 cm long intestine tube, between the jejunum and ileum, was cut (much closer to the ileum), and drawn from the mucosal compartments. The samples, with a total surface of 0.33 cm^2^, were mounted in Ussing chambers and bathed with 4 mL glucose-Krebs buffer [34]. Each chamber was equipped with a water jacket, allowing a constant temperature of 37 °C during the course of the experiments. Two 1.5% agarose electrodes were placed in each half of the chamber (150 mL 3 M KCl + 2.25 gr agarose). Tissues were continuously gassed with pure oxygen and short-circuited by an automatic DC voltage clamp device. A voltage of V = 5 mV was applied to the tissues, and the change in current (I [A]) was monitored using labchart 8 reader software (Adinstruments, Dunedin, New Zealand). The resistance (R[Ω]) was calculated using Ohm’s law, where V=∆I·R. After a period of at least 30 min, when the system reached equilibrium, 5 mM EGTA was added, and the changes in current were monitored. The value was calculated as the average of n = 7 intestinal segments obtained from three healthy female Sprague-Dawley rats (200–250 g), which were kindly provided by the laboratory of George G. Malliaras. The animals were purchased from Charles River (UK).

### 2.8. Micro-CT Scan

A micro-CT scan was conducted using a SkyScan 1172 Microtomographer (Bruker, Belgium) to detect the effect of DBSA percent on the scaffold’s pore size distribution and porosity. Datasets were reconstructed using a modified Feldkamp algorithm in NRecon software (Bruker), with output files analysed using CT-Analyser (CTan) (Bruker). Briefly, from each cylindrical sample, we selected 3 different 1.5 mm × 1.5 mm × 1.5 mm cubes in the middle of each sample and performed the analysis as shown in Figure S1b. For this analysis, we used 3 scaffolds of 0% DBSA, 2 scaffolds of 0.2%, and 2 scaffolds of 0.5% DBSA. See Supplementary Materials for details.

### 2.9. Quantitative Real-Time PCR Assay

The regulation of the mRNA expression of *ZO-1* and *αSMA* was detected in the 2D inserts and the 3D bioelectronic models. RNA was isolated using TRIzol Reagent (Thermofisher, UK). RNA isolation, cDNA synthesis and PCR were performed using Monarch Total RNA Miniprep Kit, LunaScript RT SuperMix Kit and Luna Universal qPCR Master Mix (New England BioLabs, Hitchin, UK). Real-time PCR was carried out with a QuantStudio 6 Pro real-time PCR system. Primer sequences used in this study are listed in Table S2 in the Supplementary Materials and were taken from previously published sources for *ZO-1* and *GAPDH* [35], *αSMA* [36] and *β-actin* [37]. Gene expression was analyzed with LinRegPCR (version 2021.1) [38].

### 2.10. Statistical Analysis

For the Calcium switch assay experiments, a one-way ANOVA test followed by Holm–Šídák’s multiple comparisons post hoc test was used to determine the level of significance among different experiments. For all other statistical analyses, a two-tailed Student’s *t*-test was used. Data are presented as mean, and error bars are presented as the standard deviation, and asterisks indicating significance in the figures correspond to *p* < 0.05, 0.005, 0.001, and 0.0001 shown as *, **, *** and ****, respectively. Statistical analysis was performed using GraphPad Prism (version 8.0).

## 3. Results and Discussion

### 3.1. Reconstituting the Rat Small Intestine Barrier in 2D Using Inserts

As a first step in our comparison between 2D, 3D tissue cultures and the rat ex vivo model, we established a 2D rat small intestinal cell culture using inserts. This culture included a non-tumorigenic rat fibroblast cell line (208F) and rat small intestinal epithelial cells (IEC-6) [32]. IEC-6 cells, derived from the crypt region of a healthy rat small intestine, are a well-established non-transformed epithelial line that retains characteristics of intestinal enterocytes. To evaluate barrier formation under both monoculture and co-culture conditions, we conducted TEER measurements using an EVOM device (see the Section 2 for details), along with immunofluorescence imaging targeting specific cell-type markers.

#### 3.1.1. Barrier Formation in 2D Co-Culture

**Figure 2:**
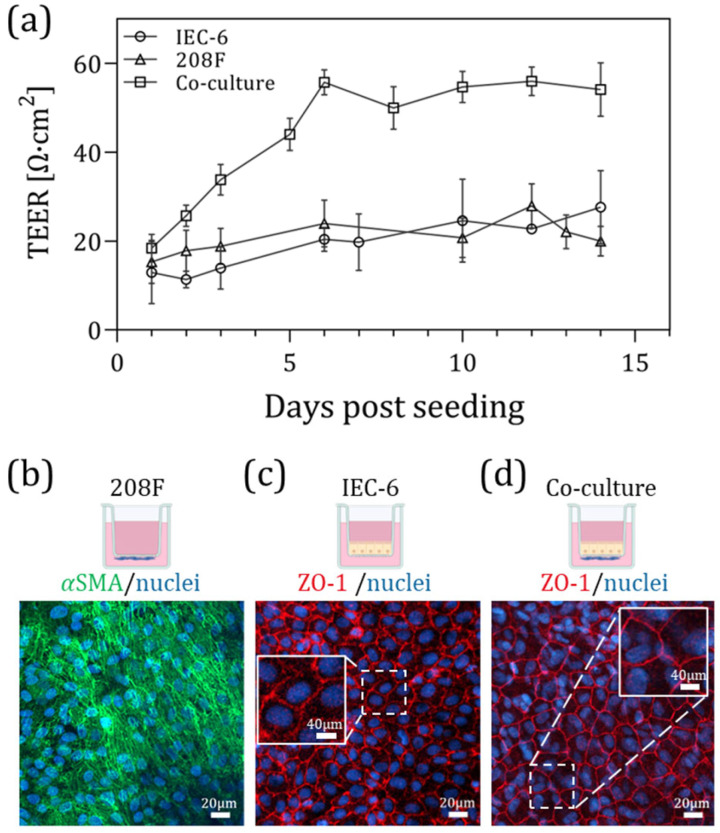
Establishment of a 2D rat small intestinal barrier model using inserts. (**a**) Barrier formation in 2D cultures measured by TEER using an EVOM device. Comparison of monocultures (208F and IEC-6) and co-cultures over 14 days. Data represent N = 4 biological replicates, with n = 3–5 technical replicates per passage. (**b**) Immunofluorescence staining of 208F cells after 14 days of culture, showing αSMA (green) and nuclei (DAPI, blue). (**c**) IEC-6 cells cultured for 14 days, stained for the tight junction protein ZO-1 (red) and nuclei (blue). (**d**) Co-culture of 208F and IEC-6 cells for 14 days, stained for ZO-1 (red) and nuclei (blue), demonstrating enhanced tight junction formation. The icons above the images were created in BioRender. Kirma, S. (2026). BioRender.com/bk6inpu.

TEER measurements were taken from day 1 post-seeding through day 14, with the TEER profile shown in Figure 2a. On day 14, cells were either used for endpoint assays or fixed for immunofluorescence staining. The TEER data indicated that the co-culture exhibited approximately 2.5-fold higher resistance compared to the monocultures, with mean TEER values of 54 ± 6 [Ω·cm^2^] for the co-culture, 27 ± 8 and 20 ± 3 [Ω·cm^2^] for the IEC-6 and 208F monocultures, respectively. These results suggest an enhanced barrier function in the co-culture system (relative to the monocultures).

These findings are consistent with previously reported TEER values for IEC-6 monoculture. For example, Fan et al. [35] reported TEER values of approximately 50 Ω·cm^2^ for IEC-6 monoculture, which is comparable to the resistance measured in our IEC-6 monoculture (27 ± 8 Ω·cm^2^). Small differences between studies may arise from variations in experimental setup, culture conditions, and TEER measurement systems.

#### 3.1.2. Tight Junction and Cell Identity Characterization by Immunofluorescence

To determine whether 208F cells expressed fibroblast-specific components, αSMA staining was performed (Figure 2b), revealing the expected structural morphology. Additionally, the expression of the tight junction protein ZO-1 was assessed to evaluate epithelial barrier formation in 2D and 3D culture (Figure 2c and Figure 2d, respectively). A closer comparison of Figure 2c,d may indicate slight differences in ZO-1 staining between the mono- and co-culture conditions, with the co-culture displaying a more uniform and continuous pattern. Non-specific αSMA staining was observed in IEC-6 monocultures, as shown in Supplementary Materials, Figure S2a; therefore, αSMA staining was not performed on the co-culture samples. It is also noted that ZO-1 staining was absent in 208F monocultures (see Supplementary Materials, Figure S2b). These findings were further supported by gene expression analysis, where RT-PCR demonstrated increased *ZO-1* and *αSMA* expression in the 3D platform compared with the 2D system (Supplementary Materials, Figure S3).

Although the 2D model provides useful insights into barrier formation, it lacks the structural complexity of the native intestinal tissue. Therefore, to better replicate the in vivo architecture, we next established a 3D rat small intestinal barrier model, using the e-transmembrane bioelectronic device to allow for monitoring of the 3D cell tissue model.

### 3.2. Establishment of a Rat Small Intestinal Barrier in a 3D Culture e-Transmembrane Device

#### 3.2.1. 3D Co-Culture Assembly

**Figure 3:**
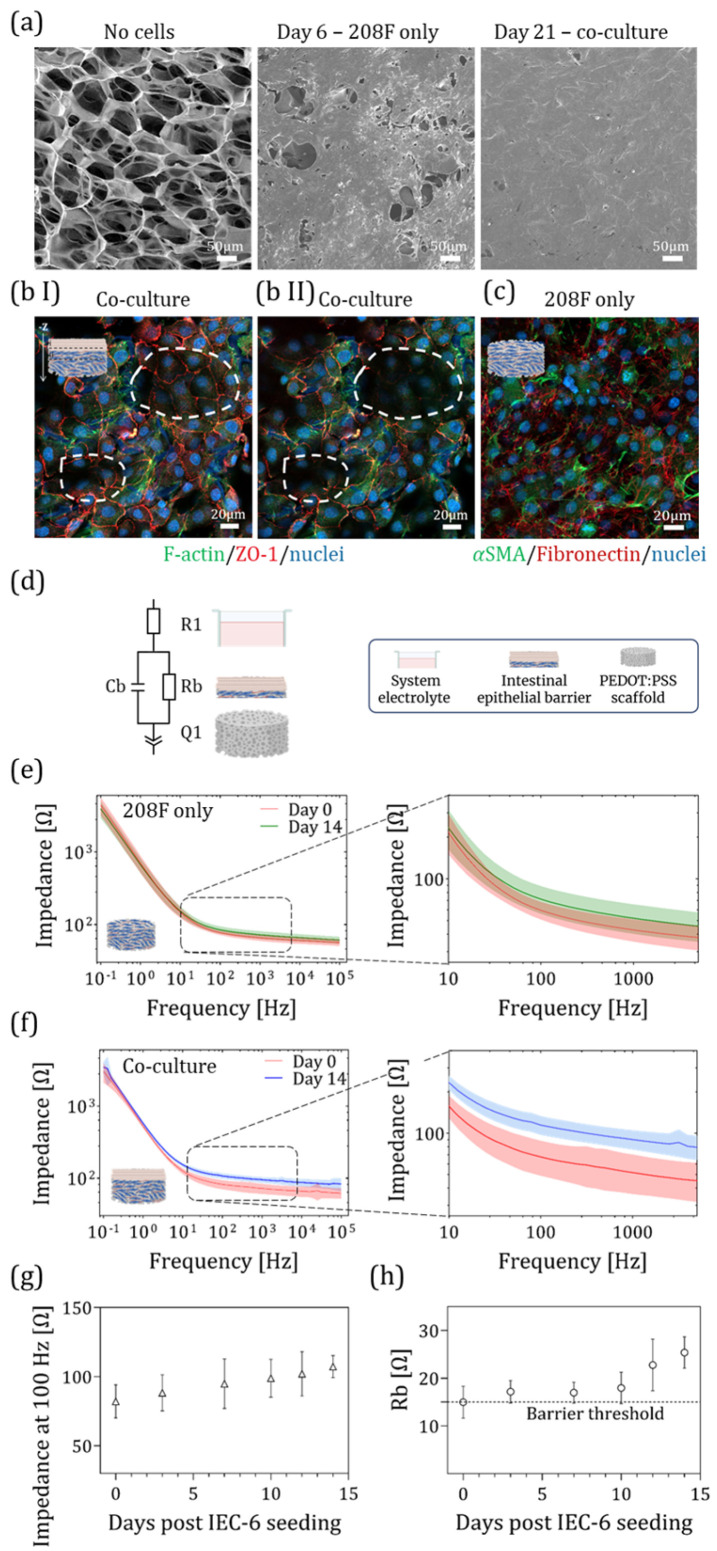
Establishment of 3D rat small intestinal barrier formation within the e-transmembrane device. (**a**) SEM imaging of the cell-free PEDOT:PSS scaffold at day 0, 208F cells within the scaffold 6 days post-seeding, and the co-culture after seeding IEC-6 cells on top of 208F cells, following 15 days of co-culture (21 days total). (**bI**,**bII**) 208F cells were cultured for 6 days, followed by the addition of IEC-6 cells on top, and co-cultured for an additional 15 days. The cells were then stained for F-actin (green), ZO-1 (red) and nuclei (blue), with Z-stack imaging performed. (**c**) 208F cells monocultured for 21 days, stained for αSMA (green), fibronectin (red) and nuclei (blue). (**d**) An equivalent circuit model of the system was used to fit the experimental data. In this model, R1 represents the resistance of the electrolyte, while Rb and Cp correspond to the resistance and capacitance of the epithelial barrier, respectively. Q1 is a constant phase element that defines the rest of the device system, mainly the PEDOT:PSS scaffold. (**e**,**f**) EIS measurements of barrier formation within the e-transmembrane device are shown for both the monoculture (N = 3 biological replicates, with n = 12 technical replicates (devices) in total) and co-culture (Data represent N = 4 biological replicates, with n = 10 technical replicates (devices) in total) conditions, comparing day 0 (corresponding to day 6 of total culture time for the co-culture) and day 14. The colored bands represent the standard deviation (SD) of the measurements, and the dark lines indicate the mean impedance across the devices. (**g**) Impedance magnitude at frequency of 100 Hz over time following IEC-6 cell seeding (N = 4, n = 10 devices). (**h**) Barrier resistance (Rb), extracted as a figure of merit using an equivalent circuit model, plotted as a function of days to monitor barrier formation during the co-culture period (N = 4, n = 10 devices). Created in BioRender. Rambam, C. (2026). BioRender.com/48duorg.

To monitor electrical signaling in an in vitro rat small intestine model, we employed a 3D electronic transmembrane device capable of EIS monitoring (Figure 1a). Originally developed in our lab to model the human gut barrier [18,19], the device was adapted for rat small intestinal tissue to better emulate in situ conditions within a PEDOT:PSS scaffold. The scaffolds we used were crosslinked with 3% (*w*/*v*) PEGDE (see Figure S1 for details). As previously found, these scaffolds are more elastic, flexible, and electrically conductive than those crosslinked with GOPS, and are more than an order of magnitude softer [39]. Briefly, following device fabrication and sterilization (for more details see Section 2), 6 × 10^5^ 208F cells were seeded on the apical side of the PEDOT:PSS scaffolds within the 3D device and maintained under immersed conditions for 5 days. During this period, the 208F cells proliferated and populated the scaffold pores, as shown in Figure 3a, which illustrates barrier formation through SEM images captured at three key time points: before cell seeding (No cells); on day 6, prior to the addition of intestinal cells; and on day 21, after full barrier development, showing complete surface coverage by IEC-6 cells. On day 6, 7 × 10^5^ IEC-6 cells were seeded on top, allowing the formation of an epithelial barrier over the next 14 days (total culture duration: 21 days). The extended co-culture period supports the development of a differentiated epithelial monolayer, which is critical for studies involving epithelial barrier function [40]. Note that there is a difference in the total culture duration between the 2D and 3D platforms, due to the additional time required for fibroblasts to proliferate and fully populate the 3D PEDOT:PSS scaffold (400 μm thick). In contrast, on the 2D insert platform, 208F cells were seeded as a monolayer 2 h before the IEC-6 seeding, resulting in a 5-day shorter culture period compared to the 3D setup.

#### 3.2.2. Cell Localization Within Scaffold by Immunofluorescence

To verify the expression and integrity of tight junction proteins in IEC-6 cells forming the epithelial barrier, we examined ZO-1 expression using Z-stack imaging (Figure 3(bI,bII)). Because of the porous structure of the scaffold, cells do not grow in a single plane. Z-stack imaging, however, revealed that the scaffold surface was covered with epithelial cells, as indicated by ZO-1 staining. Regions highlighted by the dashed circles in Figure 3(bI) showed ZO-1 staining at the surface, which was absent when moving deeper along the *Z*-axis (Figure 3(bII)). In the deeper scaffold regions (−Z direction), epithelial cells were not detected; instead, only fibroblasts were observed, indicated by the absence of ZO-1 staining (also not detected in 208F monocultures; Figure S2b) and by the presence of actin and nuclei staining (Figure S2c). To assess fibroblast-related features [41], we examined the expression of fibronectin, an ECM-associated protein, and αSMA, a typical fibroblast marker, after 21 days of monoculture within the scaffold (Figure 3c).

#### 3.2.3. EIS Monitoring of Barrier Formation

EIS measurements were used to complete the characterization of the 3D rat small intestinal platform. EIS is a non-invasive, electrochemical technique used for real-time monitoring of cells in vitro, enabling the assessment of barrier formation within the 3D device.

During tissue formation within the e-transmembrane device, the electrodes were used to monitor the impedance spectra in the frequency range between 10^5^ and 10^−1^ Hz and correlate the changes in the spectra to the cell attachment, proliferation, and barrier development. In the initial stages (day 6), the impedance spectrum resembled that of cell-free scaffolds (bode plot is presented in Figure S4a), characterized by an ohmic response at high-to-mid frequencies and dominated by the capacitive CRE response at low frequencies (Figure 3e,f). Due to the inverse proportionality of capacitive impedance with frequency and surface area (Z∝1/freq·SA), the large, capacitive, non-reactive mesh used as the CRE contributes meaningfully only at low frequencies (<100 Hz), which is similar for the capacitive contribution of the large PEDOT:PSS WE, with an impedance contribution which is inversely proportional to volume (Z∝1/freq·Vol). Thus, changes in impedance at frequencies > 100 Hz are attributable to fluctuations in the tissue impedance and the electrolyte resistance (which is nominally constant, see Supplementary Materials, Figure S4b). As the epithelial barrier formed, a marked increase in impedance magnitude appears at frequencies > 100 Hz, consistent with an increasing resistive contribution by the epithelium. EIS measurements were performed every 2–4 days (Figure 3e,f). Barrier formation was evaluated by comparing impedance magnitude at the start of co-culture (day 0 epithelial culture) to the endpoint. Colored bands represent the standard deviation (SD) of the measurements, while dark lines indicate the mean impedance magnitude. Based on comparisons to monoculture and blank devices, we defined a threshold for barrier formation as an impedance of ≥95 Ω at 100 Hz on day 14. Only samples meeting this criterion were included in the analysis. Failures in barrier formation were mainly due to technical variability between devices, defects in the PEDOT:PSS scaffolds, or similar factors.

#### 3.2.4. Impedance Response of Mono- Versus Co-Culture

Figure 3e shows impedance magnitude, including a zoomed-in view of the mid-range frequency (~100–1000 Hz, where the cell barrier is most often dominant) for the monoculture condition (208F cells only; N = 3 biological replicates, n = 12 technical replicates). Phase angle data are shown in Supplementary Materials, Figure S4c. The monoculture fibroblast model exhibits a small increase in mid-frequency impedance compared to the initial values before cell seeding. For the co-culture devices (Figure 3f), a distinct increase in overall impedance magnitude is observed in the mid-range frequency (N = 4 biological replicates, n = 10 technical replicates). Phase angle data are shown in Supplementary Materials, Figure S4d.

Note that day 0 of the co-culture corresponds to day 6 of total culture time. As shown in Figure 3f, there is a significant separation (with *p*-value < 0.034 for frequency >0.31 Hz, when using a paired, two-tailed Student’s *t*-test) between day 0 and day 14 impedance curves in the co-culture, indicating a resistive contribution by the developing epithelium. This is consistent with the development of an epithelial barrier, with a resistive paracellular current pathway. In contrast, the monoculture condition shows closely aligned curves at both time points, suggesting no such barrier development. These last results (monoculture condition) are nearly equivalent to the cell-free control condition, presented in Supplementary Materials, Figure S4a (n = 5 devices), indicating a small but negligible resistive contribution by the fibroblast cell-mass under monoculture conditions.

Direct comparison of impedance or resistance values between different 3D experimental platforms is challenging, as each system is typically designed and optimized with different device configurations, electrode geometries, and measurement setups that require platform-specific modeling. Therefore, absolute electrical values should be interpreted within the context of each platform rather than directly compared across different 3D models reported in the literature. Instead, the temporal evolution of the impedance signal within each system provides more meaningful information regarding barrier formation. In our system, Figure 3e,f demonstrates a clear increase in mid-frequency impedance over time in the co-culture condition, whereas the monoculture condition shows minimal impedance changes between time points. Together, these results indicate resistive contributions from the developing epithelial layer, consistent with the formation of a functional epithelial barrier in the co-culture model.

#### 3.2.5. Barrier Resistance Quantification

For further evaluation, the impedance magnitude at 100 Hz was extracted from the co-culture devices (N = 4, n = 10 devices), revealing a gradual increase over time following IEC-6 cell seeding (Figure 3g). The mean impedance value was significantly increased from 82.08 ± 12.00 Ω on day 0 to 107.26 ± 8.04 Ω on day 14 (with a *p*-value of =1.5∙10^−4^ when using paired, two-tailed Student’s *t*-test). In order to disambiguate between changes in the epithelial contribution and potential changes in the electrodes and electrolyte, the barrier resistance (Rb), which reflects the resistive contribution of epithelial cells to the impedance, was derived from the data. Using a complex non-linear least squares regression, the equivalent circuit model was fitted to the experimental data as described in Supplementary Materials, Figure 3d (for more details, see Section 2 and [19]) and the Rb value plotted over time to monitor barrier formation during the co-culture period (Figure 3h; N = 4, n = 10 devices). The mean Rb value increased from 14.97 ± 3.36 Ω on day 0 to 25.37 ± 3.30 Ω on day 14, with a *p*-value of 0.0053 using a *t*-test (due to technical failures, not all chips yielded measurements at both time points. Therefore, comparisons between day 0 and day 14 were performed using an unpaired two-tailed Student’s *t*-test). Furthermore, the Rb value and the electrolyte resistance are shown to be uncorrelated (ρ=0.192) with the electrolyte resistance largely constant and the two parameter values significantly distinct at day 14 with a *p*-value of 0.027 (differences were analyzed using an unpaired two-tailed *t*-test, see Supplementary Materials, Figure S4b). Indicating that the fluctuations in impedance magnitude at mid and high frequency are primarily due to epithelial barrier formation. Additionally, the independence of the two resistance parameters in the model lends credibility to the model selection. Both the raw impedance magnitude at 100 Hz and the extracted barrier resistance increase monotonically over time. This is consistent with an epithelial proliferating towards confluence. Further, the barrier resistance increases at a greater rate from day 10 to day 14, which is consistent with barrier formation and tight junction expression following confluence. Together, these data demonstrate the model’s ability to recapitulate and measure epithelial tissue function and the dependence thereof on the co-culture. The same dataset was used to generate Figure 3e–g, with only devices reaching ≥95 Ω at 100 Hz on day 14 (post IEC-6 seeding) included in the analysis. Consistent with the functional and structural characterization, gene expression analysis demonstrated increased *ZO-1* and *αSMA* expression in the 3D platform compared with the 2D culture system (Supplementary Materials, Figure S3).

### 3.3. Assessment of Barrier Integrity and EGTA-Induced Disruption via Resistance Measurements

As the final step in validating our in vitro 3D bioelectronics platform and comparing it with both the traditional 2D in vitro insert model and the ex vivo condition of rat small intestinal tissue, we performed a calcium switch assay to assess the barrier’s response to 5 mM EGTA after 30 min of exposure. We then evaluated barrier recovery, 30 min after washing, using barrier resistance measurements. Each model was monitored using its specific method: for the 2D in vitro model, barrier disruption and recovery were monitored using an EVOM device; for the 3D in vitro model, barrier impedance was measured using EIS with a potentiostat; and for the ex vivo tissue, measurements were conducted using a Ussing chamber. For the Ussing chamber experiments, a small segment of rat intestinal tissue was used, taken from the region between the jejunum and ileum, closer to the ileum (the experimental setup is shown in Supplementary Materials, Figure S5).

**Figure 4:**
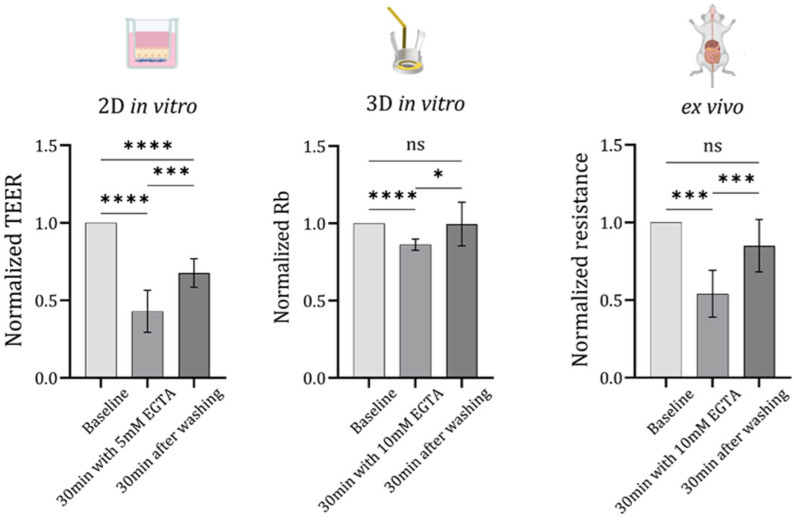
Comparison of barrier integrity before and after EGTA-induced disruption, assessed by resistance measurements. Once barrier formation was achieved (after 14 days for the 2D model and 21 days for the 3D model) and following gut tissue extraction for the ex vivo condition, barrier resistance was measured at three time points: before 5 mM EGTA addition (baseline), after 30 min of EGTA incubation, and 30 min after washing with media. Statistical analysis was performed using a one-way ANOVA with Holm–Šídák’s multiple comparisons test in Prism software version 10 (* *p* < 0.05, ** *p* < 0.005, *** *p* < 0.001, **** *p* < 0.0001). For the 2D in vitro experiments, data represent N = 3 biological replicates with a total of n = 11 technical replicates; for the 3D in vitro model, N = 3 biological replicates with n = 9 technical replicates (devices); and for the ex vivo experiments, tissue from 3 rats was used, with n = 9 technical replicates (gut segments). All incubations were performed at 37 °C in a humidified atmosphere containing 5% CO_2_. The icons above the graphs were created in BioRender. Kirma, S. (2026). BioRender.com/a3pwwmy.

Note that, as previously reported for the e-transmembrane platform, the figure of merit Rb reflects the resistance of the epithelial barrier and serves as an indicator of its integrity [19]. However, Rb is not directly comparable to conventional TEER values; EIS (and consequently Rb) is a small signal linearization measurement while TEER is calculated from the large-signal, non-linear system at steady state (square wave transient analysis). In the Ussing chamber measurement, a small signal perturbation is used (similar to EIS) at a single frequency, where a four-wire configuration (Kelvin resistance measurement) is used to measure the resistivity of the tissue film, which completely separates the two electrode (current and source) pairs (see Section 2 for details). While all three utilize Ohm’s law to infer resistivity (or more generally impedance), the non-linearity of the system limits absolute comparison of electrical readouts across platforms. To enable comparison between the in vitro 2D, 3D, and ex vivo models, resistance measurements were normalized to the baseline value obtained for each system prior to EGTA addition, allowing for relative changes in barrier integrity to be compared across the different experimental configurations. This normalization allows for a meaningful comparison of barrier dynamics (biological phenomena correlated in time) across distinct measurement platforms despite differences in absolute values (Figure 4).

Briefly, once barrier formation was achieved (after 14 days for the 2D model and 21 days for the 3D model), and following gut tissue extraction for the ex vivo condition, barrier resistance was measured at three time points: before EGTA addition (baseline), after 30 min incubation with 5 mM EGTA, and 30 min after washing with media.

The results are presented in Figure 4 as normalized resistance values, where resistance measurements after EGTA treatment and subsequent washing were normalized to the baseline measurement for each method. The results show significant barrier disruption across all three platforms following 30 min of exposure to 5 mM EGTA (with normalized mean TEER value of 0.83 ± 0.09 [a.u] (2D in vitro), normalized mean Rb value of 0.99 ± 0.13 [a.u] (3D in vitro), and normalized mean resistance value of 0.85 ± 0.16 [a.u] (ex vivo)). During the recovery phase, only the 3D in vitro and ex vivo platforms demonstrated tissue recovery, whereas in the 2D model, a significant difference remained between the baseline and recovery measurements (with normalized mean TEER value of 0.68 ± 0.13 [a.u] (2D in vitro), normalized mean Rb value of 0.83 ± 0.03 [a.u] (3D in vitro), and normalized mean resistance value of 0.54 ± 0.14 [a.u] (ex vivo)). The observed differences may be explained by diffusion effects, which occur more rapidly in the 2D platform. Consequently, EGTA can reach all cells more efficiently in the 2D insert platform than in the 3D or ex vivo systems, potentially affecting the results.

These findings suggest that the 3D platform follows a trend similar to the ex vivo assay, indicating that the 3D model more closely mimics ex vivo conditions compared to the 2D model. Furthermore, these results provide functional validation for the 3D bioelectronic device, reinforcing its potential as a predictive tool for drug screening and intestinal barrier studies. Nevertheless, several limitations should be acknowledged. The current platform demonstrates reduced sensitivity in detecting low-resistance epithelial barriers, limiting its ability to accurately resolve subtle changes in weaker or partially disrupted tissues. Ongoing work is focused on improving device sensitivity through optimization of the electrochemical configuration, including the implementation of more advanced counter and reference electrode systems. In addition, the present model is primarily based on epithelial–fibroblast co-culture and does not yet incorporate the full cellular complexity of the native small intestine. Future optimization may include integration of additional relevant cell types, such as endothelial cells and other intestinal cell populations, to better recapitulate tissue architecture and intercellular crosstalk.

It is worth noting that we also tested 10 mM EGTA in the 2D EVOM experiments; however, this concentration proved too harsh for the cells, which were unable to recover. Therefore, a concentration of 5 mM was selected for all subsequent experiments.

## 4. Conclusions

In the present study, we validated a 3D bioelectronic platform for modeling the rat small intestine, motivated by the need for more physiologically relevant in vitro models. Through integrated bioelectronic measurements, including TEER and barrier responses to EGTA, we demonstrated the formation of a structurally and functionally relevant epithelial barrier. These measurements indicated that the 3D model more closely mimics native rat intestinal tissue ex vivo compared with traditional 2D cultures. This validation confirms the platform’s utility for applications such as drug screening, disease modeling, and microbiota–host interaction studies, providing a reliable and complementary tool to conventional animal experiments.

The TEER values obtained in our models were consistent with previously reported measurements for IEC-6 monocultures. Minor differences between studies may be attributed to variations in experimental setup, measurement systems, and operator handling. Overall, the electrical properties of our 3D model reflect the low-resistance physiological characteristics of small intestinal epithelium, as was reported in the literature, further supporting the functional relevance of the system and highlighting the advantages of integrating bioelectronic monitoring within 3D tissue models.

Additional gene expression analysis of *ZO-1* and *αSMA* (presented in the Supplementary Materials) further supported the enhanced barrier-related characteristics observed in the 3D platform compared with the 2D system.

Taken together, these results demonstrate the successful establishment of a bioelectronic 3D intestinal barrier model capable of recapitulating key structural, molecular, and electrical features of native tissue. This platform provides a promising tool for studying intestinal physiology and pathology, with potential applications in personalized medicine, toxicology testing, and advanced in vitro disease modeling. Future work will focus on expanding the platform to incorporate additional cell types, microbiota components, and physiologically relevant stimuli, thereby further improving model complexity and predictive capability.

## Data Availability

Data available in a publicly accessible repository. The original data presented in the study are openly available in Apollo-University of Cambridge Repository at: https://doi.org/10.17863/CAM.126557.

## Supplementary Materials

The following supporting information can be downloaded at: https://www.mdpi.com/article/10.3390/bioengineering13030349/s1, Figure S1: PEDOT:PSS scaffold morphology and electrical characterization; Table S1: Maximum pore size distribution; Figure S2: Control and co-culture immunofluorescence analysis of IEC-6 and 208F cells; Figure S3: RT-PCR gene expression analysis *ZO-1* and *αSMA* in 2D and 3D co-cultures; Table S2: Primer sequences used in real-time PCR; Figure S4: EIS measurements within the e-Transmembrane device; Figure S5: Overview of the ex vivo experimental setup.

## Abbreviations

The following abbreviations are used in this manuscript:

PEDOT:PSS	poly(3,4-ethylenedioxythiophene):poly(styrenesulfonate)
IEC-6	intestinal epithelial cells
EIS	electrochemical impedance spectroscopy
EGTA	ethylene glycol tetraacetic acid
e-transmembrane	electronic transmembrane
iPS cells	induced pluripotent stem cells
DBSA	sodium dodecylbenzenesulfonate
GOPS	3-glycidyloxypropyl)trimethoxysilane
PEGDE	poly(ethylene glycol) diglycidyl ether
TEER	transepithelial electrical resistance
RT	room temperature
SEM	scanning electron microscopy
CE	counter electrode
WE	working electrode
RE	reference electrode
RT-PCR	real-time PCR
