# Supplementary material for: Comparison of 2D, 3D In Vitro, and Ex Vivo Platforms for Modeling the Rat Small Intestine
Source: Bioengineering (Basel). 2026 Mar 17;13(3):349. doi: 10.3390/bioengineering13030349 (PMC13024708; doi:10.3390/bioengineering13030349)
Supplement: Supplementary file 1 [file bioengineering-13-00349-s001.zip › bioengineering-4168735-supplementary.pdf]

Comparison of 2D, 3D In Vitro, and Ex Vivo Platforms for Modeling the Rat Small Intestine

Supplementary Information

Shani Elias-Kirma [1*], Reece McCoy [1], Douglas van Niekerk [1], Verena Stoeger [1], Sophie Oldroyd [1], Emma Sumner [1†], Achilleas Savva [1,2] and Róisín M. Owens [1*]

[1] Department of Chemical Engineering and Biotechnology, University of Cambridge, Philippa Fawcett Drive, Cambridge CB3 0AS, UK
[2] Department of Microelectronics, Faculty of Electrical Engineering, Mathematics and Computer Science, Delft University of Technology, Mekelweg 4, 2628 CD Delft, The Netherlands
[†] This manuscript is dedicated to the memory of Emma Sumner, who tragically passed away during its preparation.
[*] Correspondence: rmo37@cam.ac.uk; se446@cantab.ac.uk

<u>*Calibration of DBSA Concentration in the Scaffold*</u>

To verify that the scaffold met the experimental requirements, such as achieving a suitable pore size for cell seeding and sensitivity to slight changes in epithelial barrier electrical impedance, the concentration of DBSA added to the PEDOT: PSS mixture was calibrated. DBSA is a commonly used additive in PEDOT:PSS scaffolds, owing to its role as a surfactant and its ability to enhance conductivity[1–3].

Scaffolds fabricated with varying DBSA concentrations (0%, 0.2%, and 0.5% w/v) were characterized by micro-CT scanning, SEM imaging, and EIS measurements (Figure S1). All other fabrication conditions were kept constant, including 3% (w/v) PEGDE as the crosslinker (see Materials and Methods). Morphological differences were evaluated by SEM (Figure S1a) and quantified through micro-CT analysis, which provided pore size distributions (Figure S1b; Table S1). Scaffolds containing 0.2% and 0.5% DBSA displayed similar pore distribution profiles, with maximum pore sizes of 83.38 ± 0.93 μm and 77.42 ± 1.27 μm, respectively. In contrast, scaffolds without DBSA showed a smaller maximum pore size of 59.55 ± 1.23 μm, which is more favorable for cell seeding.

To assess the effect of DBSA concentration on electrical impedance, EIS signals of cell-free e-transmembrane devices (assembled with medium but without cells) were monitored over 12 days. Average results are presented in Figures S1c–e. Impedance magnitude was analyzed at 100 Hz, where the epithelial barrier is

expected to be detected during co-culture with IEC-6 and 208F cells (mid-range frequency ~100–1000 Hz). Devices without DBSA consistently exhibited lower average impedance compared to those with 0.2% DBSA (Figure S1c). Furthermore, Bode plots at day 0 (baseline) and day 12 confirmed this difference: devices without DBSA (blue) displayed lower impedance values than those with 0.2% DBSA (red) (Figures S1d, e). The shaded areas represent the standard deviation

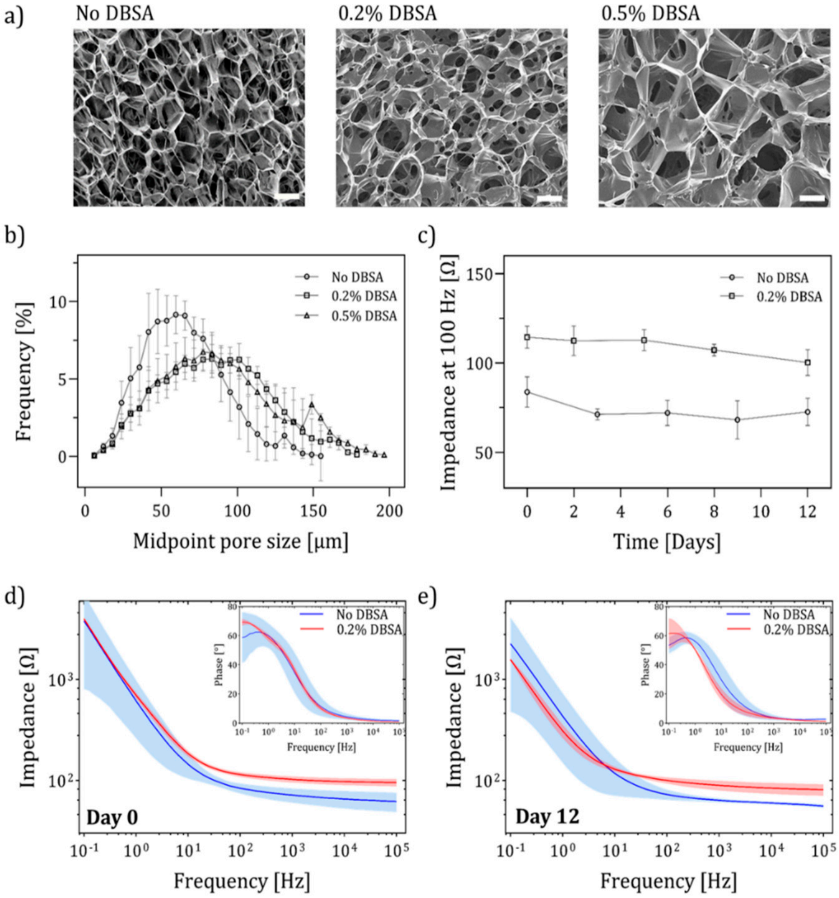

Figure S1. PEDOT: PSS scaffold morphology and electrical characterization. (a) SEM imaging depicting the morphological differences across the three DBSA concentrations. (b) Micro-CT scan analysis of PEDOT: PSS + 3% PEGDE scaffolds with varying DBSA concentrations: No DBSA (N=3), 0.2% (N=2), and 0.5% (N=2) (w/v) DBSA, with n=3 for each scaffold analysis. (b) (c) Impedance magnitude at 100 Hz for cell-free devices monitored over 12 days, with No DBSA (N=5 devices) and 0.2% DBSA (N=3 devices). (d, e) Bode plot showing the impedance magnitude and Phase of the devices on day 0 and day 12. The colored bands represent the standard deviation (SD) of the measurements, while the dark blue and dark red lines indicate the mean impedance and phase for devices with No DBSA (N=5 devices) and 0.2% DBSA, respectively (N=3 devices).

(SD) of the measurements, while the dark lines indicate mean impedance and phase.

Based on these characterizations, a PEDOT: PSS scaffold with 3% (w/v) PEGDE as the crosslinker and no DBSA was selected. This composition provides pore sizes suitable for cell seeding and supports barrier formation when co-cultured with IEC-6 and 208F cells.

Table S1. Maximum pore size distribution

	Pore Size (μm)	SD (μm)	Percentage of Total Pores (%)
No DBSA	59.55	1.23	9.2
0.2% DBSA	83.93	0.93	6.3
0.5% DBSA	77.42	1.27	6.8

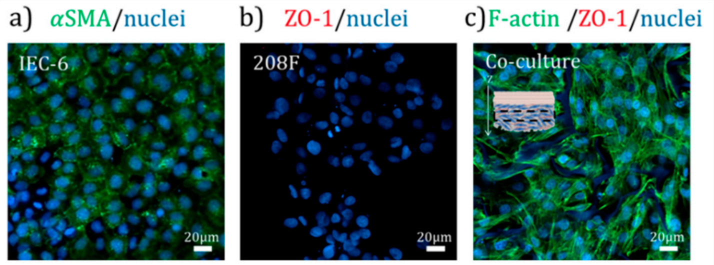

Figure S2. Control and co-culture immunofluorescence analysis of IEC-6 and 208F cells. (a) Immunofluorescence staining of IEC-6 cells after 2 days of culture on a well plate, showing non-specific staining of αSMA (green) and nuclei (DAPI, blue). (b) Immunofluorescence staining of 208F cells after 2 days of culture on a well plate, showing absence of ZO-1 (red) staining and nuclei (DAPI, blue). (c) 208F cells cultured for 6 days within the e-Transmembrane device, followed by the addition of IEC-6 cells on top and co-cultured for an additional 15 days. Cells were then stained for F-actin (green), ZO-1 (red), and nuclei (blue). Z-stack imaging performed in the -Z direction revealed that only fibroblasts were present in the deeper scaffold regions, as indicated by the lack of ZO-1 staining and the presence of actin and nuclei staining.

To further strengthen the functional characterization of epithelial barrier formation, we assessed the gene expression levels of ZO-1 and αSMA using qRT-PCR analysis (Figure S3). The results demonstrate a significant upregulation of both markers in the 3D platform compared with the 2D culture system. Specifically, ZO-1, a key tight junction protein, and αSMA, a marker associated with fibroblast cells, showed significantly higher expression levels in the 3D model relative to the housekeeping gene. These findings are consistent with the immunofluorescence results demonstrating ZO-1 junctional localization, as well as with the electrical resistance measurements. Together, these complementary morphological, molecular, and functional data further support the successful

formation of an epithelial barrier in the 3D system. This analysis was performed to provide additional molecular validation of barrier integrity rather than to conduct a comprehensive epithelial differentiation profiling.

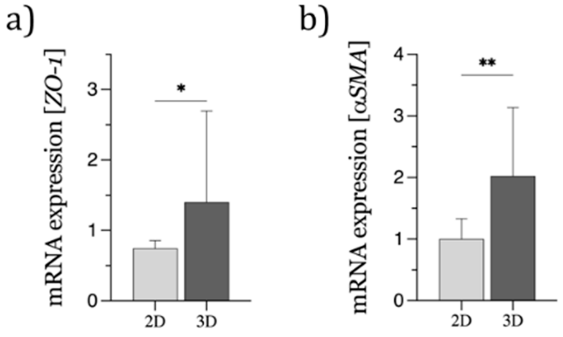

Figure S3. RT-PCR gene expression analysis of (a) ZO-1 and (b) αSMA in 2D and 3D co-cultures.

Table S2. Primer sequences used in real-time PCR

Gene	Species		Primer (5′-3′)
ZO-1	Rat	Forward	CCACCTCGCACGTATCACAAGC
		Reverse	GGCAATGACACTCCTTCGTCTCTG
αSMA	Rat	Forward	GCTATTCAGGCTGTGCTGTC
		Reverse	GGTAGTCGGTGAGATCTCGG
GAPDH	Rat	Forward	CCCTCTGGAAAGCTGTGG
		Reverse	GCTTCACCACCTTCTTGATGT
β-actin	Rat	Forward	CACCAGTTCGCCATGGATGACGAT
		Reverse	TCTCTTGCTCTGGGCCTCGTCG

The barrier resistance and electrolyte resistance (zeroth order regression parameter) are plotted in Figure S3b. The electrolyte resistance can be seen to be largely time independent (relative to fluctuation in the barrier resistance), indicating that the only time varying resistivity in the system is Rb. Further, the Pearson correlation between the two parameters is calculated as ϱ=0.19. The poor correlation between the parameters implies that the ill-posed regression does not require further regularization to disambiguate between the two variables. Taken together, the implication is that measured changes in Rb are likely to be well correlated to fluctuations in the tissue paracellular resistance.

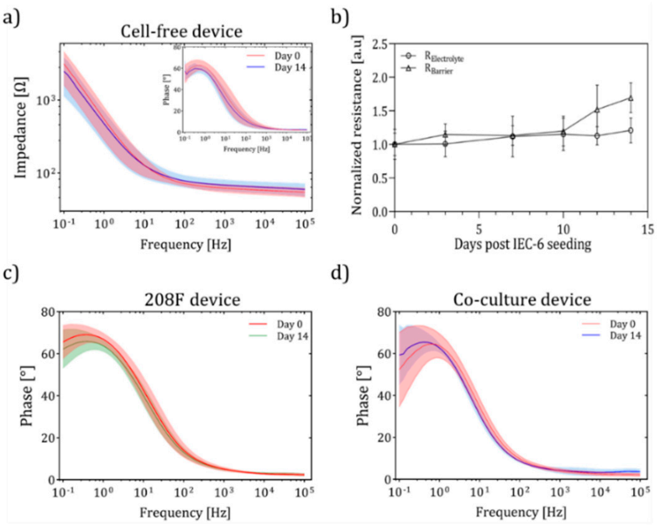

Figure S4. **EIS measurements within the e-Transmembrane device.** Colored bands represent the standard deviation (SD) of the measurements, and dark lines indicate the mean impedance or phase angle. (a) bode plot of cell-free devices on day 0 and 14 of culture (n = 5 devices). (b) Plot of the fitted electrolyte and barrier resistances over time (scaled to the day 0 value). (c) Phase angle plot of 208F mono-culture devices N = 3 biological replicates, n = 12 technical replicates. (d) Phase angle plot of Co-culture devices N = 4 biological replicates, n = 10 technical replicates.

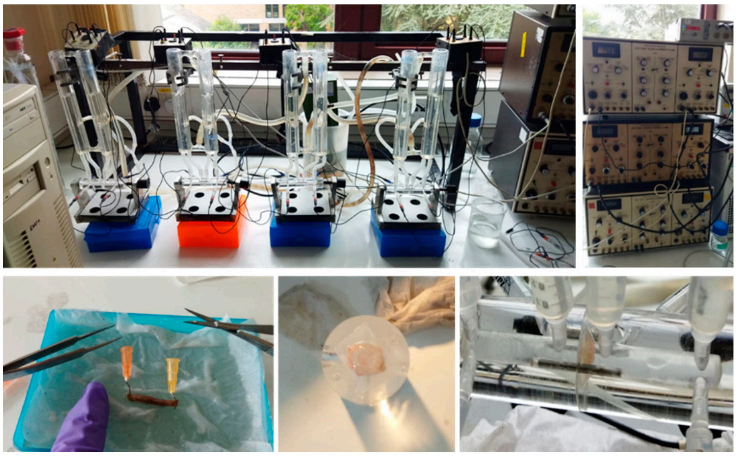

Figure S5. Overview of the *ex vivo* experimental setup. The Ussing chamber experimental setup used for the *ex vivo* investigation of barrier disruption in rat small intestine tissue.
